# Downregulation of TRAF2 Mediates NIK-Induced Pancreatic Cancer Cell Proliferation and Tumorigenicity

**DOI:** 10.1371/journal.pone.0053676

**Published:** 2013-01-03

**Authors:** Heike Döppler, Geou-Yarh Liou, Peter Storz

**Affiliations:** Department of Cancer Biology, Mayo Clinic Comprehensive Cancer Center, Mayo Clinic, Jacksonville, Florida, United States of America; Technische Universität München, Germany

## Abstract

**Background:**

Increased levels of NF-κB are hallmarks of pancreatic ductal adenocarcinoma (PDAC) and both classical and alternative NF-κB activation pathways have been implicated.

**Methodology/Principal Findings:**

Here we show that activation of the alternative pathway is a source for the high basal NF-κB activity in PDAC cell lines. Increased activity of the p52/RelB NF-κB complex is mediated through stabilization and activation of NF-κB-inducing kinase (NIK). We identify proteasomal downregulation of TNF receptor-associated factor 2 (TRAF2) as a mechanism by which levels of active NIK are increased in PDAC cell lines. Such upregulation of NIK expression and activity levels relays to increased proliferation and anchorage-independent growth, but not migration or survival of PDAC cells.

**Conclusions/Significance:**

Rapid growth is one characteristic of pancreatic cancer. Our data indicates that the TRAF2/NIK/NF-κB2 pathway regulates PDAC cell tumorigenicity and could be a valuable target for therapy of this cancer.

## Introduction

The transcription factors of the NF-κB (nuclear factor κ-light-chain-enhancer of activated B cells) family are upregulated in many human cancers [1]. NF-κB has roles in all hallmarks of carcinogenesis or cancer progression, including protection from cell death, increase of cell proliferation, cell motility and metastasis, tumor inflammation and angiogenesis [1]. In addition, tumor cells often acquire resistance to anticancer drugs (chemoresistance) by upregulating NF-κB signaling [2].

NF-κB transcription factor complexes are formed by homo- or heterodimers of the subunits p65 (RelA), RelB, c-Rel, p50 or p52 [3]. RelA/p50 dimers represent the classical (canonical) NF-κB1 and RelB/p52 dimers the alternative (non-canonical) NF-κB2 complex [4]. Both the alternative and classical NF-κB activation pathways rely on the IκB kinase (IKK) complex that is composed of IKKα, IKKβ and NEMO/IKKγ. IKKβ and NEMO/IKKγ mediate the activation of the canonical NF-κB1 pathway, in which IKKα has no essential role. In contrast, activation of the alternative NF-κB2 pathway requires IKKα, but not IKKβ and NEMO [5]. It also involves NF-κB-inducing kinase (NIK) as a direct upstream kinase for IKKα [4]. Once activated by NIK, IKKα induces the processing of NF-κB2/p100 to p52.

In absence of a stimulus, NIK is rapidly degraded and this depends on its association with TNF receptor-associated factor 3 (TRAF3). Binding to TRAF3 recruits NIK to the TRAF2/cIAP1/cIAP2 ligase complex [6], [7]. Cellular inhibitor of apoptosis proteins (cIAPs) are ubiquitin ligases that can promote the ubiquitination and proteasomal degradation of themselves, as well as their binding partners TRAF2 and TRAF3 [8], [9]. Both cIAPs also mediate K48-linked polyubiquitination of NIK, resulting in its proteasomal degradation [7]. In stimulated cells (i.e. upon CD40 receptor engagement), TRAF2/cIAP1/cIAP2/TRAF3 complexes are recruited to the receptor and TRAF2 induces ubiquitination and degradation of TRAF3 [10]. Since TRAF3 levels decrease, newly synthesized NIK is stabilized and active because it no longer can interact with the TRAF2/cIAP1/cIAP2 complex [6].

In pancreatic ductal adenocarcinoma cancer (PDAC), NF-κB levels are increased in cancer cell lines as well as patient samples and mediate cell proliferation and resistance to chemotherapy [11], [12], [13]. Increased NF-κB activity in PDAC is due to both the canonical and alternative activation pathways [14], [15]. Since so far no genetic alterations for TRAFs, cIAP or NIK were described for this cancer, the mechanisms by which the alternative pathway is upregulated are largely unknown for PDAC.

Here we show that in PDAC cell lines TRAF2 protein levels are downregulated and that this is the mechanism by which stabilization of NIK is achieved to induce activation of the alternative NF-κB pathway. We further show that NIK activity relays to increased cell proliferation and anchorage-independent growth. Rapid growth is one hallmark of pancreatic cancer and our data indicates that the TRAF2/NIK/NF-κB2 pathway may be a valuable target for therapy of this cancer.

## Results

### NIK Expression and Activity are Increased in PDAC Cell Lines

**Figure 1:**
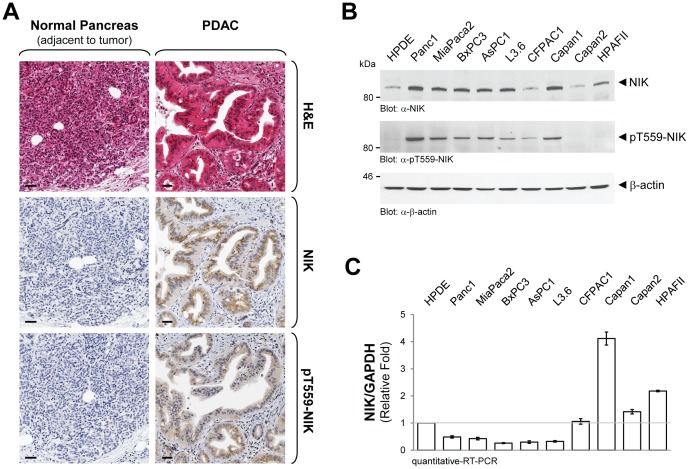
NIK expression and activity are increased in PDAC and PDAC cell lines. **A:** Human tissue samples of normal pancreas and PDAC were immunohistochemically-stained for total NIK (anti-NIK) or active NIK (anti-pT599-NIK) expression, or subjected to H&E staining. The bar indicates 50 µm. **B:** Cell lysates of indicated PDAC cell lines or HPDE cells were normalized to 0.5 mg/ml and then subjected to SDS-PAGE. Samples were transferred to nitrocellulose and analyzed by Western blot for expression of NIK (anti-NIK) or NIK activity (anti-pT559-NIK). Staining for β-actin (anti- β-actin) served as loading control. **C:** Samples of mRNA of indicated cell lines were subjected to quantitative RT-PCR directed against NIK. Samples were normalized to GAPDH.

Active NIK is overexpressed in human samples of PDAC as compared to normal pancreatic tissue (**Fig. 1A**). This promoted us to analyze a panel of nine established PDAC cell lines, as well as human pancreatic ductal epithelial (HPDE) cells that served as normal control for expression and activity of NIK. In most PDAC cells lines that were analyzed, NIK expression was increased as compared to normal HPDE cells (**Fig. 1B**, top panel). Increased expression correlated with increased activity as determined with a phospho-specific antibody (anti-pT559-NIK) that recognizes NIK phosphorylation at its activation loop (**Fig. 1B**, middle panel). Of all PDAC cell lines analyzed, only two, Capan2 and HPAFII, did not show increased activity of NIK. Using quantitative RT-PCR, we next tested if increased NIK mRNA expression could be the reason for the increased protein levels. Although we observed variations in NIK mRNA expression between the different cell lines, an obvious correlation with its expression at the protein level was not noted (**Fig. 1C**). This indicated that in PDAC cell lines increased NIK expression is not achieved by increased transcription, but rather by mechanisms that stabilize protein levels.

### TRAF2 Expression is Downregulated in PDAC Cell Lines

**Figure 2:**
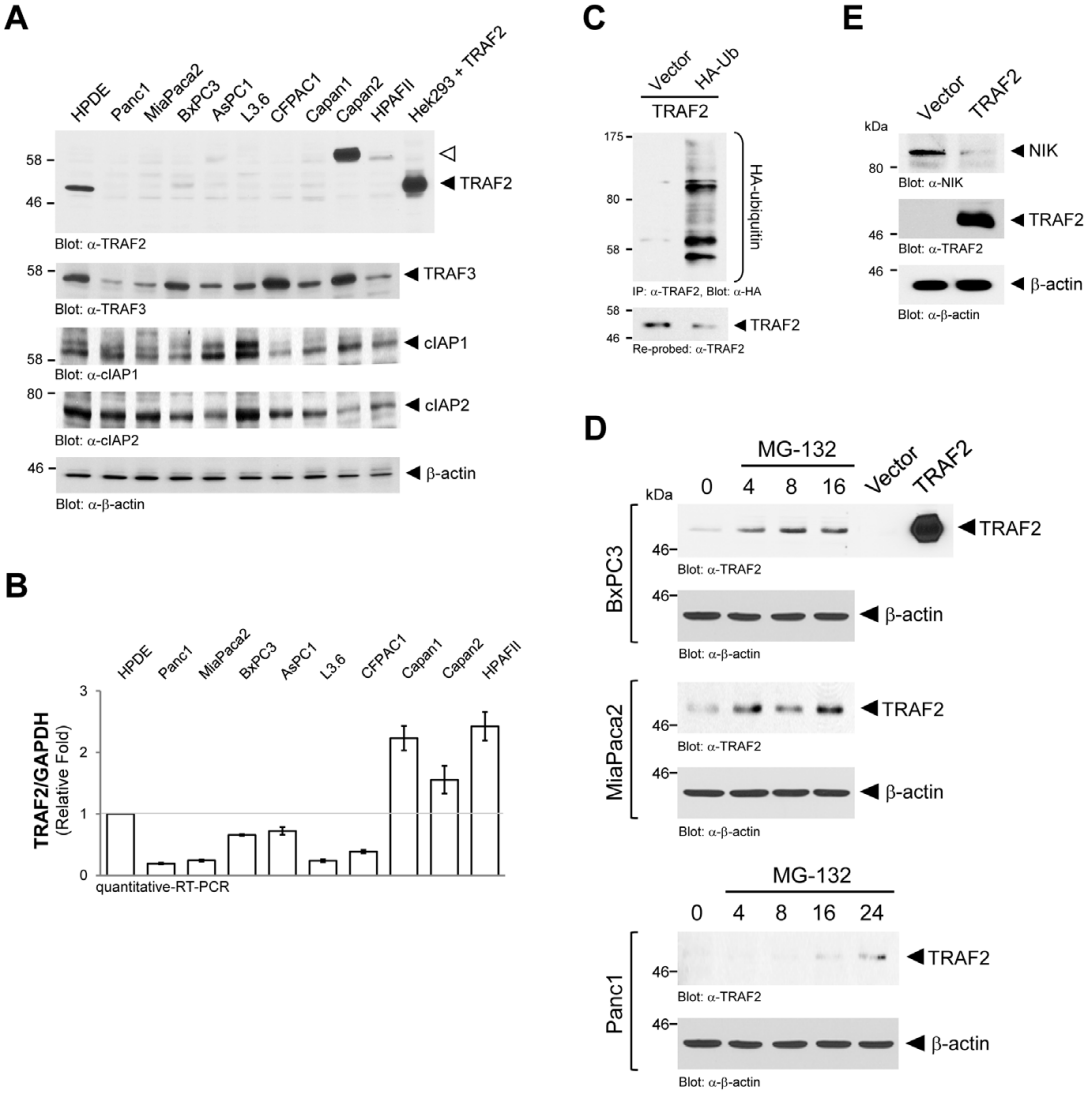
TRAF2 expression is downregulated in PDAC cell lines. **A:** Cell lysates of indicated cells were normalized to 0.5 mg/ml and then 20 µg were subjected to SDS-PAGE. Samples were transferred to nitrocellulose and analyzed by Western blot for expression of TRAF2 (anti-TRAF2), TRAF3 (anti-TRAF3), cIAP1 (anti-cIAP1), cIAP2 (anti-cIAP2) or β-actin (anti-β-actin; loading control). TRAF2 overexpressed in Hek293 cells served as an additional molecular weight control. **B:** Samples of mRNA of indicated cell lines were subjected to quantitative RT-PCR directed against TRAF2. Samples were normalized to GAPDH. **C:** Panc1 cells (5×10^5^ cells, 6 cm dishes) were co-transfected with TRAF2 and vector control or HA-ubiquitin. After 24 hours cells were lysed, TRAF2 immunoprecipitated (anti-TRAF2) and analyzed by immunoblotting for ubiquitination of TRAF2 (anti-HA). Blots were re-probed for TRAF2 (anti-TRAF2). **D:** Indicated cell lines were treated with MG-132 (20 µM) for 0, 4, 8, 16 or 24 hours. Cells were lysed and analyzed for expression of endogenous TRAF2 (anti-TRAF2) or β-actin (anti-β-actin; loading control. TRAF2 overexpressed in Hek293 cells served as positive control. **E:** Panc1 cells (5×10^5^ cells, 6 cm dishes) were transfected with vector control or TRAF2 as indicated. After 24 hours cell lysates were analyzed by Western blotting for expression of NIK (anti-NIK), overexpressed TRAF2 (anti-TRAF2) or β-actin (anti-β-actin) as loading control.

We next tested if NIK is regulated posttranslationally. In absence of an activating signal, NIK was shown to be downregulated in its expression by interaction with the TRAF2, TRAF3 and cIAP1/2 complex and subsequent ubiquitination [15], [16]. Of this regulatory complex for NIK, TRAF3 and cIAP1/2 are abundantly expressed in all PDAC cell lines, as well as normal HPDE control cells (**Fig. 2A**). TRAF2, however, was only expressed in HPDE cells, but not, or very little expressed in seven of nine PDAC cell lines. In Capan1 and HPAFII cells TRAF2 expression was detected, but at an unusual molecular weight of approximately 65 kDa (**Fig. 2A**, **Fig. S1**, white arrow). Loss of TRAF2 expression was observed in all PDAC cell lines that showed increased NIK activity (compare **Fig. 1A** and **Fig. 2A**). Interestingly, TRAF2 mRNA was abundant in all PDAC cell lines (**Fig. 2B**), indicating that its expression is also not regulated at transcriptional levels. Since TRAF2 expression levels can be regulated by ubiquitination, we next tested if its loss in PDAC cell lines is due to ubiquitination and subsequent proteasomal degradation. When re-expressed in Panc1 cells, TRAF2 was ubiquitinated (**Fig. 2C**), whereas no ubiquitination was detected when ectopic TRAF2 was expressed in HPDE cells (not shown). Treatment of PDAC cell lines with MG-132, a proteasome inhibitor, restored endogenous TRAF2 levels within 4 to 24 hours, dependent on the cell line (**Fig. 2D**). Moreover, ectopic re-expression of TRAF2 in Panc1 led to a decrease in NIK expression, further indicating that increased NIK levels are mediated by downregulation of TRAF2 in these cells (**Fig. 2E**). Taken together, this suggests that one mechanism of how basal high NIK expression and activity levels are regulated in the majority of PDAC cell lines is by ubiquitination and proteasomal degradation of TRAF2, resulting in increased NIK stability and activity.

### NIK Mediates Basal NF-κB Activity in PDAC Cell Lines

**Figure 3:**
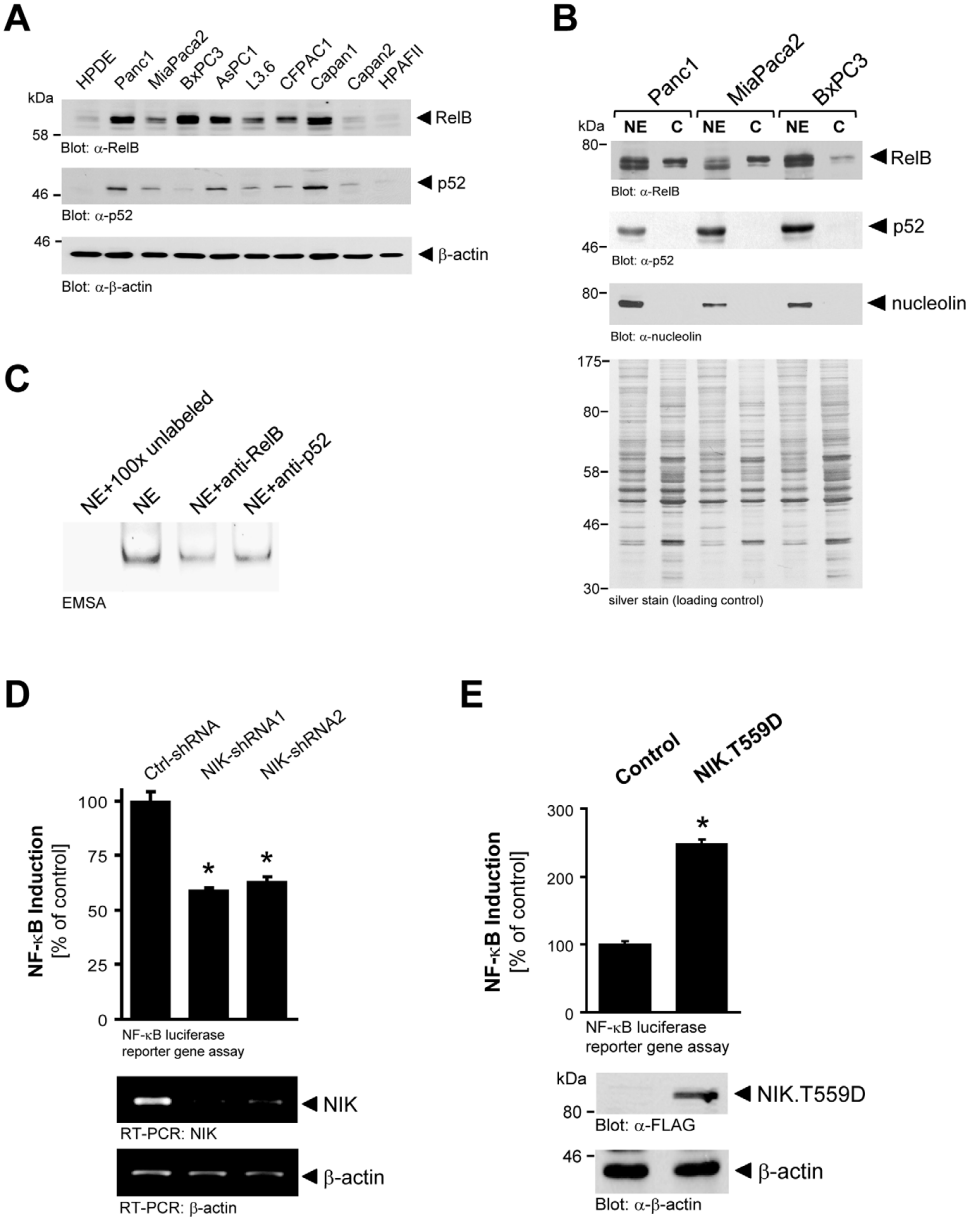
NIK mediates basal NF-κB activity in PDAC cell lines. **A:** Cell lysates of indicated cells were normalized to 0.5 mg/ml and then 20 µg were subjected to SDS-PAGE. Samples were transferred to nitrocellulose and analyzed by Western blot for expression of RelB (anti-RelB), p52 (anti-p52) or β-actin (anti-β-actin; loading control). **B:** Nuclear extracts and cytosolic fractions of indicated cell lines were analyzed by Western blotting for RelB and p52 and additionally for p65 and p50 (see **Supplemental Figure S2**). Immunostaining for nucleolin served as a marker for nuclear extracts. Silver staining of the lysates served as loading control. **C:** Nuclear extracts of Panc1 cells were incubated with anti-RelB or anti-p52 antibodies as indicated and then subjected to DNA binding activity analysis using EMSA. 100x unlabeled oligonucleotide served as a specificity control. **D:** Panc1 cells expressing control (scrambled) shRNA or NIK-shRNA (two different sequences, NIK-shRNA1 or NIK-shRNA2) were additionally transfected with NF-κB-luciferase and renilla luciferase reporters. 16 hours after transfection reporter gene luciferase assays were performed. Knockdown of NIK was controlled with RT-PCR analysis. RT-PCR for β-actin mRNA served as control. The asterisks indicate statistical significance. **E:** Panc1 cells were transfected with NF-κB-luciferase and renilla luciferase reporters as well as vector control or a NIK.T599D mutant, as indicated. 16 hours after transfection reporter gene luciferase assays were performed. Cell lysates were analyzed for expressed active NIK using Western blot and antibodies directed against FLAG-tagged NIK.T559D (anti-FLAG) or β-actin (anti-β-actin) as a loading control. The asterisk indicates statistical significance.

Once activated NIK can induce the non-canonical NF-κB activation pathway, by inducing the processing of NF-κB2/p100 to p52. In total cell lysates of PDAC cell lines, increased levels of p52 correlated with increased NIK expression and activity indicating that active NIK is also functional (compare **Fig. 3A** and **Fig. 1B**). We also observed increased expression of RelB, a *bona fide* binding partner for p52 in the alternative NF-κB pathway, indicating that increased NIK activity leads to formation of RelB/p52 NF-κB complexes. Indeed, both components of this complex were detected in nuclei of PDAC cell lines (**Fig. 3B**) and EMSA supershift analysis showed that both have DNA binding activity (**Fig. 3C**). Of note, nuclear extracts also contained p65 and p50 (**Fig. S2**). Using luciferase reporter gene assays we next tested if NIK-mediated induction of NF-κB2 relays to increased basal levels of NF-κB. When Panc1 cells were lentivirally infected with control shRNA or two specific NIK-shRNA sequences (NIK-shRNA1 and NIK-shRNA2) and then transfected with NF-κB-luciferase gene reporter, we observed a significant decrease in NF-κB activity when NIK was downregulated in its expression (**Fig. 3D**). In contrast, when cells were transfected with an active allele of NIK (NIK.T559D) basal NF-κB levels were increased (**Fig. 3E**).

### NIK is a Critical Regulator of Transformed Growth in PDAC Cells

**Figure 4:**
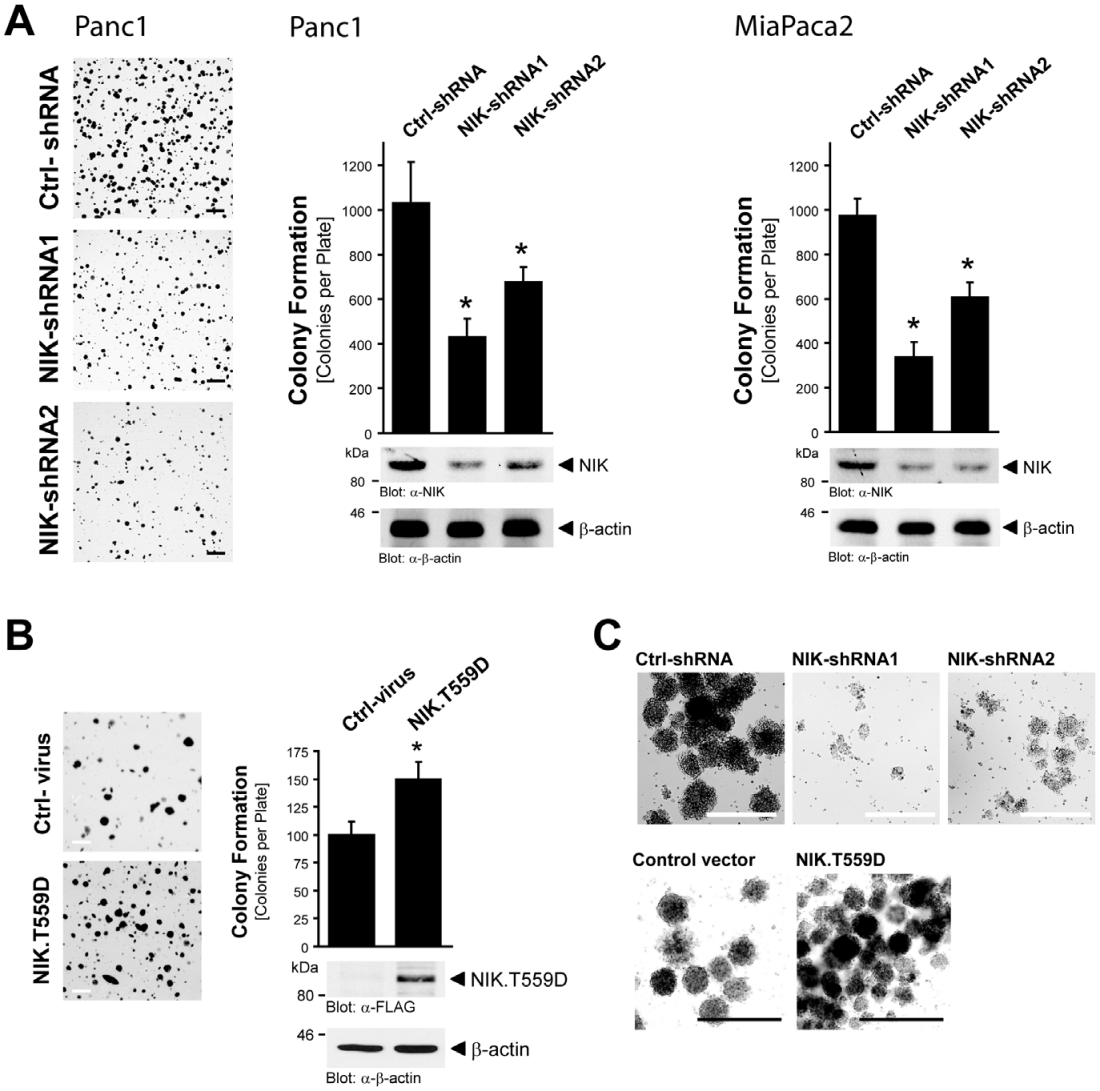
NIK is a critical regulator of transformed growth in PDAC cells. **A:** Panc1 or MiaPaca2 cells (5×10^5^ cells, 6 cm dishes) stably expressing control (scrambled) shRNA or NIK-shRNA (two different sequences, NIK-shRNA1 or NIK-shRNA2) were subjected to soft agar colony formation assays. A fraction of the transfected cells were collected and analyzed for NIK expression using Western blot analysis and antibodies directed against NIK (anti-NIK) or β-actin (anti-β-actin) as loading control. The asterisks indicate statistical significance. Scale bars represent 1 mm. **B:** Panc1 cells (5×10^5^ cells, 6 cm dishes) were lentivirally infected with control virus or virus for expression of constitutively active NIK (NIK.T559D mutant) and subjected to soft agar colony formation assays. Before seeding a fraction of the cells were lysed and analyzed for expressed active NIK using Western blot and antibodies directed against FLAG-tagged NIK.T559D (anti-FLAG) or β-actin (anti-β-actin) as a loading control. The asterisk indicates statistical significance. Scale bars represent 1 mm. **C:** Panc1 cells (5×10^5^ cells, 6 cm dishes) stably expressing control (scrambled) shRNA, NIK-shRNA1 or NIK-shRNA2 (top row), or cells lentivirally infected with control virus or NIK.T559D mutant (bottom row) were seeded in 3D Matrigel culture. At days 10 (shRNA cells) or 14 (NIK.T559D expressing cells) after seeding colony growth was analyzed by ImagePro. The bar represents 500 µm.

Next we assessed the requirement of NIK in PDAC cell transformed growth. Therefore, we first established Panc1 and MiaPaca2 cell lines stably expressing either NIK-shRNA or control scrambled shRNA. We then utilized these cell lines to determine anchorage independent growth in softagar colony formation assays. The knockdown of NIK in Panc1 and MiaPaca2 cells significantly decreased anchorage-independent growth and this correlated with the NIK protein expression levels in cells (**Fig. 4A**). Ectopic expression of a constitutively active NIK allele increased anchorage-independent growth and colony formation in Panc1 cells (**Fig. 4B**). Similar results on cell growth were obtained with above reverse genetics (**Fig. 4C**, top row) or ectopic overexpression (**Fig. 4C**, bottom row) approaches, when Panc1 cells were grown in 3-dimensional (3D) cell culture in Matrigel instead of softagar. Our results indicate that NIK is necessary and sufficient to mediate tumorigenic growth of PDAC cell lines.

### TRAF2/NIK Signaling Regulates PDAC Cell Proliferation

**Figure 5:**
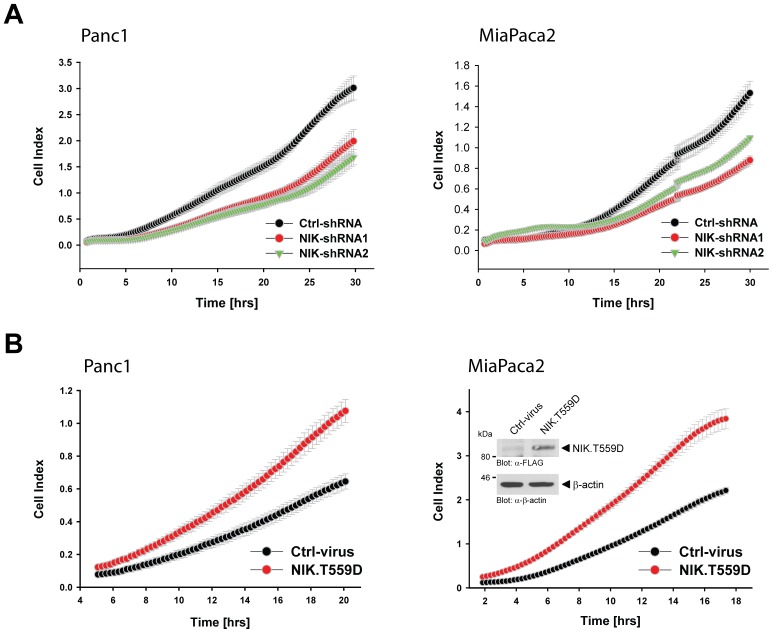
NIK regulates PDAC cell proliferation. **A:** Panc1 or MiaPaca2 cells (5×10^5^ cells, 6cm dish) stably expressing control (scrambled) shRNA or NIK-shRNA (two different sequences, NIK-shRNA1 or NIK-shRNA2) were seeded in E-plates and after attachment cell proliferation was continuously monitored in real-time for indicated times using an xCELLigence RTCA DP instrument. Error bars (gray) represent three experiments. Control analysis of knockdown of NIK for both cell lines is shown in Figure 4A. **B:** Panc1 or MiaPaca2 cells (5×10^5^ cells, 6 cm dishes) were lentivirally infected with control virus or virus for expression of constitutively active NIK (NIK.T559D mutant). The next day media was changed and after 48 hours cells were seeded in E-plates and after attachment cell proliferation was continuously monitored in real-time for indicated times using an xCELLigence RTCA DP instrument. Error bars (gray) represent three experiments. A fraction of the transfected cells were lysed and analyzed for expressed active NIK using Western blot and antibodies directed against FLAG-tagged NIK.T559D (anti-FLAG) or β-actin (anti-β-actin) as a loading control (shown for MiaPaca2). The control blots for the Panc-1 cell line are shown in **Fig. 4B**.

We next tested if NIK affects proliferation, motility and chemoresistance of PDAC cells. Therefore we utilized our Panc1 and MiaPaca2 cell lines expressing NIK-shRNA or control scrambled shRNA and performed real-time proliferation assays over a time period of 30 hours. In Panc1 cells, knockdown of NIK with two different shRNA sequences substantially decreased cell proliferation to 62−/+1.1 percent for sequence 1 and 51−/+1 percent for sequence 2 relative to the control (100%) at the endpoint of analysis. In MiaPaca2 cells, knockdown of NIK decreased cell proliferation to 49−/+0.7 percent for sequence 1 and 63−/+1.7 percent for sequence 2 at the endpoint of analysis (**Fig. 5A**). In addition, Panc1 and MiaPaca2 cell lines expressing the constitutively-active NIK.T559D mutant showed increased cell proliferation to 138−/+3.7 percent (Panc1) and 166−/+2.7 percent (MiaPaca2) relative to the control (100%) at the endpoint of analysis (**Fig. 5B**). We did not observe increased sensitivity to cell death when cells were stably transfected with NIK-shRNA (not shown). Moreover, the knockdown of NIK did not sensitize PDAC cell lines to chemotherapeutics-induced cell death. To test this we compared cell lines for their responsiveness to Gemcitabine- and 5-Fluorouracil (5-FU) (**Fig. S3**). However when compared to the untreated control, the knockdown cell lines showed a decreased signal in MTT, due to their decreased potential to proliferate. Finally, we tested if the potential to migrate or invade is altered by NIK expression, but found no significant difference in cell migration or invasion in cells depleted from NIK or cells overexpressing active NIK (**Fig. S4**). This indicates that basal activity of NIK in PDAC cells exclusively affects proliferation.

**Figure 6:**
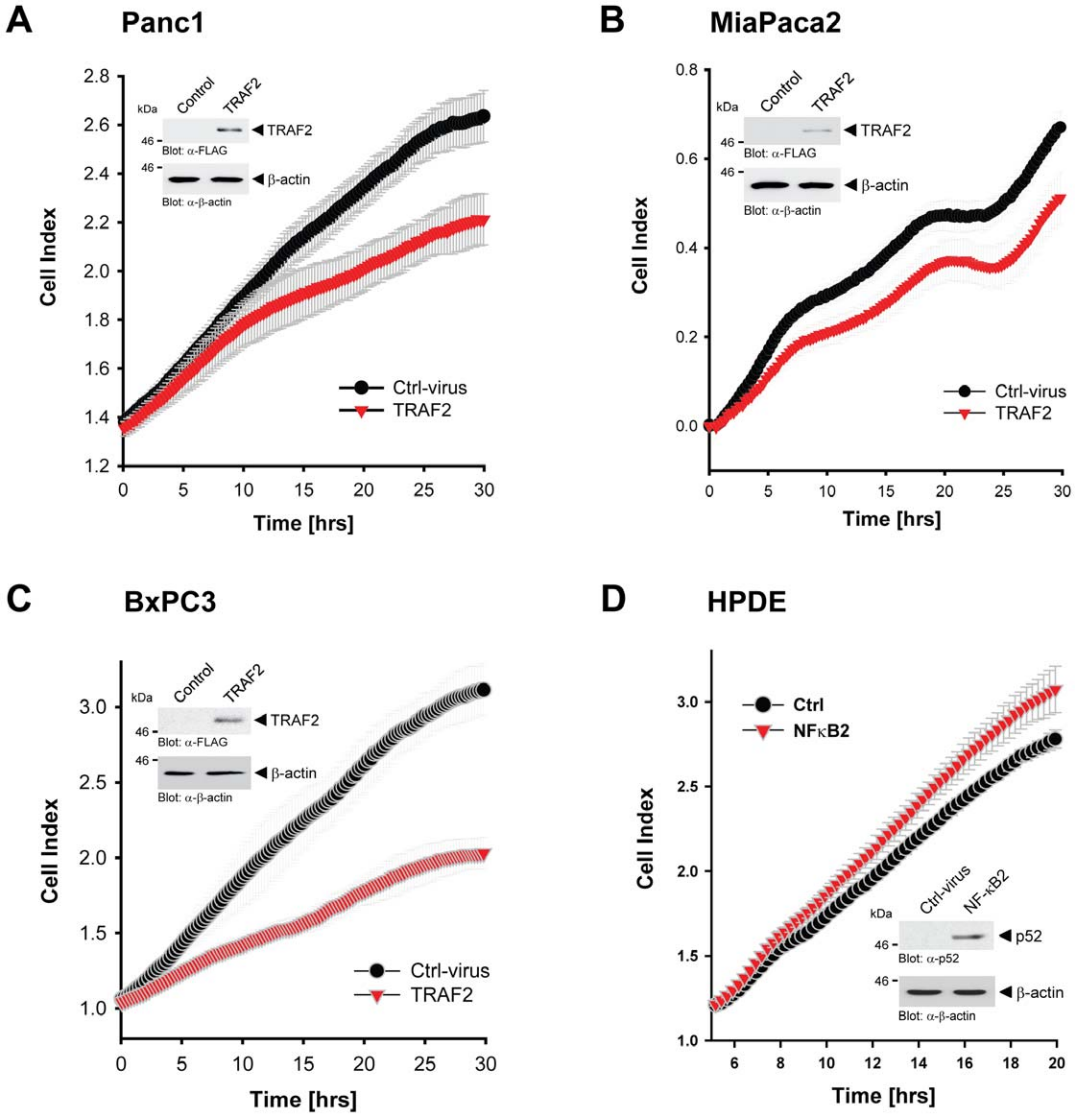
TRAF2 and NF-κB2 regulate PDAC cell proliferation. **A–C:** Indicated cell lines (5×10^5^ cells, 6 cm dishes) were infected with control or TRAF2 adenovirus. After 48 hours cells were seeded in E-Plates and after attachment cell proliferation was continuously monitored in real-time for 30 hours using an xCELLigence RTCA DP instrument. Error bars (gray) represent three experiments. A fraction of the transfected cells were lysed and analyzed by Western blot for TRAF2 or β-actin (loading control). **D:** HPDE cells (5×10^5^ cells, 6 cm dishes) were infected with control or NF-κB2/p100 adenovirus. After 48 hours cells were seeded in E-Plates and after attachment cell proliferation was continuously monitored in real-time for 20 hours using an xCELLigence RTCA DP instrument. Error bars (gray) represent three experiments. A fraction of the transfected cells were lysed and analyzed by Western blot for processed, active NF-κB2 using antibodies directed against p52 (anti-p52) or β-actin (anti-β-actin) as a loading control.

Since our previous data indicate that NIK stability and activity in PDAC cell lines are achieved by proteasomal downregulation of TRAF2, we next tested if the re-expression of TRAF2 can decrease cell proliferation. Ectopic expression of TRAF2 decreased cell proliferation of Panc1, MiaPaca2 and BxPC3 cells. Dependent on the cell line, cell proliferation was decreased to 68−/+1 percent (Panc1), 82−/+1 percent (MiaPaca2), or 48−/+0.5 percent (BxPC3) at the endpoint of analysis (30 hours). Eventually, we tested if activation of the alternative NF-κB2 pathway can mediate cell proliferation of normal pancreatic ductal cells. Therefore, we infected normal HPDE cells with NF-κB2/p100 and analyzed proliferation by real-time measurement. After expression of NF-κB2/p100, we detected its active product p52 in HPDE cells, correlating with slightly increased cell proliferation to 107−/+1.2 percent relative to the control (100%) at the endpoint of analysis (**Fig. 6D**).

### Correlation of *in vitro* Data with Clinical Data

**Figure 7:**
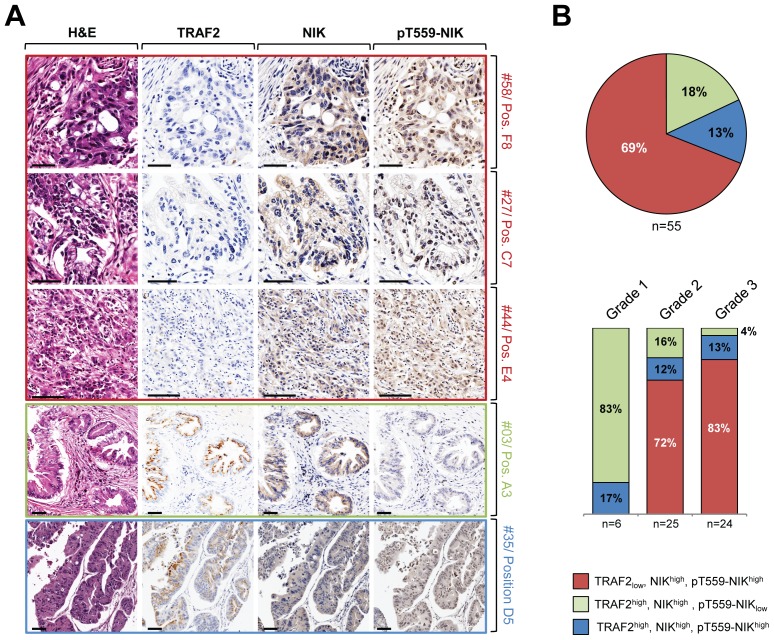
Expression of TRAF2, NIK and NIK activity in human samples for pancreatic cancer. **A:** Tissue microarrays (TMAs) including 10 normal pancreatic tissue samples and 55 pancreatic ductal adenocarinoma were H&E stained or analyzed for the expression of TRAF2 (anti-TRAF2), NIK (anti-NIK) or active NIK (anti-pT559-NIK). Representative pictures of tumor tissues are depicted. Numbers indicate the position of the tissue on the TMA. The bar indicates 50 µm. **B:** Analysis of correlation of TRAF2, NIK, and pT559-NIK in n = 55 human samples for pancreatic addnocarcinoma. Top pie graph shows percentage of cells with low TRAF2 expression and high expression of NIK and pT559-NIK in red, percentage of cells with high TRAF2 and NIK expression and low expression of pT559-NIK in green, and percentage of cells with high TRAF2, NIK and pT559-NIK expression in blue. Bottom bar graphs show percentage of these groups in grade1, grade 2, and grade 3 tumors.

Next we determined if a similar reverse correlation between NIK expression and activity and TRAF2 downregulation occurs in human samples of pancreatic adenocarcinoma. Out of 55 human PDAC samples analyzed, 69% showed decreased TRAF2 expression correlating with increased NIK levels and increased NIK activity (**Fig. 7A**, samples #58, #27 and #44 in red frame and **Fig. 7B** top pie graph red area). However, 18% of samples showed upregulation of TRAF2 and NIK levels, but no or very little NIK activity (**Fig. 7A**, sample #30 and **Fig. 7B** pie graph green area). Additional 13% of all samples showed upregulation of all three molecules (**Fig. 7A**, sample #35 and **Fig. 7B** pie graph blue area). No correlation of TRAF2/NIK levels with either age, sex, stage of tumor or TNM state were observed However, there was a correlation between tumor grade and TRAF2/NIK levels. Well differentiated tumors of grade 1 showed either high TRAF 2 and NIK and low pT559-NIK levels, or had all three upregulated. Tumors of this grade appear normal and are not growing rapidly. In contrast more than 70% of grade 2 tumors (moderately differentiated) and more than 80% of grade 3 tumors (poorly differentiated) showed downregulation of TRAF2 correlating with increased NIK levels and activity. Tumor cells of these grades appear abnormal and tend to grow and spread more aggressively, correlating with our *in vitro* data showing that above described TRAF2/NIK signaling mechanism can mediate pancreatic tumor cell proliferation.

## Discussion

Increased basal NF-κB activity was detected in human pancreatic cancer samples, as well as PDAC cell lines [11]. NIK has been identified as a key regulator of NF-κB in many cancers [17]. In the present work we show that active NIK is expressed in patient tissue for pancreatic cancer and PDAC cell lines (**Fig. 1**). We identify downregulation of TRAF2 through ubiquitination as a mechanism by which this is achieved (**Fig. 2**). In PDAC cells increased stability and activity of NIK relays to increased activation of the alternative NF-κB pathway (NF-κB2; p52/RelB). This mediates cell proliferation and anchorage-independent growth (**Figs. 4** **,** **5** **,** **6**), all hallmarks of pancreatic cancer. Our data are supported by previous work of Schneider et al., showing that the p52/RelB NF-κB complex can regulate G1- to S-phase progression in PDAC cell lines [18].

In many cancers NF-κB activation in malignant cells occurs in response to inflammatory cytokines [19]. For example, TNFα-induced activation of the canonical NF-κB pathway has been implicated as potential cause for increased chemoresistance [2]. However, there are examples of cancers where stabilization of NIK and resulting activation of NF-κB is achieved by intrinsic mutations. In approximately 20% of multiple myeloma cases loss-of-function mutations for TRAF2, TRAF3 and cIAP1/2 have been identified in patients, leading to NIK-induced activation of both the canonical and alternative NF-κB pathways to regulate cell growth and survival [20], [21].

In the alternative NF-κB activation pathway, in absence of a stimulus, NIK associates with the TRAF3/TRAF2/cIAP1/cIAP2 complex that targets it for degradation. Upon receptor stimulation the TRAF2/TRAF3/cIAP1/cIAP2 complex is clustered at the receptor and TRAF2 activation leads to cIAP1/2 and/or TRAF3 degradation [6]. This stabilizes cytosolic NIK and allows downstream signaling to activate IKKα and NF-κB2/p100 [6], [10]. But NIK also can be stabilized by other mechanisms. For example, TWEAK, a known inducer of the alternative NF-κB pathway triggers the degradation of cIAP1/2 and this results in NIK stabilization and NF-κB2/p100 processing [7]. Here we describe an additional and different activation mechanism that occurs in PDAC cell lines. Our data suggest permanent downregulation of TRAF2 protein in PDAC cell lines under normal growth conditions, leading to a stabilization of NIK expression and activation of the alternative NF-κB pathway.

Out of nine PDAC cell lines tested, seven were negative for TRAF2 protein expression and two expressed TRAF2 at an unusual molecular weight of approximately 65 kDa instead of 53 kDa (**Fig. 2A**). It is possible that this larger TRAF2 protein represents a splice form or a fusion protein. However, increased size does not seem to impact TRAF2 function towards NIK since in both cell lines, similar to HPDE control cells, NIK protein expression was low (**Fig. 1B**). The nature and function of this TRAF2 isoform will need more in-depth investigation in future work. It will also be interesting to determine if TRAF2 protein of increased molecular weight exist in patients.

**Figure 8:**
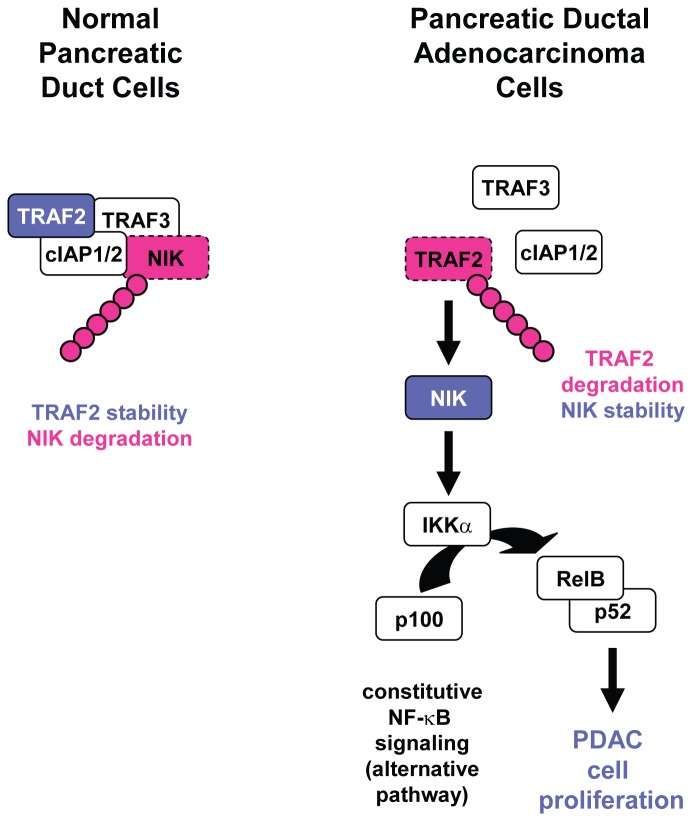
Proposed mechanism of NIK/TRAF2 signaling in PDAC cell lines. Proposed mechanism of how NIK activity is maintained in PDAC cell lines. In normal pancreatic ductal cells TRAF2 is expressed and forms a degradation complex for NIK with TRAF3, cIAP1/2. This leads to ubiquitination and degradation of NIK. In PDAC cells, TRAF2 is degraded by ubiquitination. This prevents formation of a NIK degradation complex and NIK remains expressed. NIK signals to the non-canonical IKK complex and IKKα mediates activation of NF-κB2 and formation of RelB/p52 dimers. Net effect of such signaling is an increase in pancreatic cancer cell proliferation.

We found that loss of TRAF2 expression is mediated through ubiquitination of the protein (**Figs. 2C, 2D**). No significant changes in expression of cIAP1/2 or TRAF3 were detected (**Fig. 2A**). It was shown before that the ubiquitin ligases cIAP1 and cIAP2 promote the proteasomal degradation of their binding partners TRAF2 and TRAF3 [8], [9]. Thus observed loss of TRAF2 expression could be due to presence of cIAP1/2. At this point it is unclear why in order to activate the alternative NF-κB pathway in PDAC, downregulation of TRAF2 is preferred to downregulation of TRAF3 or cIAPs instead. An explanation for such regulation may be that cIAPs previously were shown to be of importance for tumor cell survival [22]. Downregulation of TRAF2 may prevent stimulus-dependent degradation of cIAP1/2 [6], therefore allowing constitutive survival signaling through IAPs. Additionally, TRAF2 is an important scaffolding link between cIAPs and NIK and loss of TRAF2 in the majority of PDAC cell lines tested, may uncouple cIAP1/2 mediated survival signaling from their function to deplete cells from NIK. Thus with this mechanism PDAC cells may maintain both high capacity to survive, as well as high proliferation rates (**Fig. 8**).

To bolster our mechanistic *in vitro* analysis, we analyzed 55 human samples of PDAC (grades 1–3) and found that approximately 69% showed decreased expression of TRAF2 and increased levels of NIK and pT559-NIK (**Fig. 7**) similar as we had observed it in pancreatic cancer cell lines (**Figs. 1** and **2**). However, approximately 31% of samples analyzed showed high TRAF2 expression similar as previously described by Trauzold *et al.* [23]. Importantly, in all these samples, NIK expression was also upregulated. It is possible that this subset of patient samples express the higher molecular weight variant of TRAF2 that we had observed occurring in some PDAC cell lines (i.e. HPAFII). This will need further refinement in future studies. When separating tumors by grade, we found that tumors with high TRAF 2 levels were well differentiated tumors of grade 1. Tumors of this grade are not growing rapidly, whereas tumors of grades 2 and 3 are moderate or poorly differentiated, respectively, and tend to grow and spread more aggressively. This increase in aggressive growth and decreased TRAF2 expression in grade 2 and 3 tumors correlates with our *in vitro* data showing that above described TRAF2/NIK signaling mechanism can mediate pancreatic tumor cell proliferation.

In summary, our results identify proteasomal downregulation of TRAF2 as a mechanism of how NIK stability can be regulated in PDAC. Targeting this pathway to decrease the proliferation of cancer cells may represent a new strategy for therapy in PDAC. For example, the alternative NF-κB activation pathway is sensitive to the proteasome inhibitor bortezomib [20], [21]. Therefore, one potential combination therapy approach would be to combine bortezomib with cIAP inhibitors and/or apoptosis-inducing drugs such as Gemcitabine.

## Materials and Methods

### Ethics Statement

TMAs were bought from US Biomax (Rockville, MD), or biospecimens were obtained from the Mayo Clinic SPORE for Pancreatic Cancer Tissue Core under an approved Mayo Clinic Institutional Review Board protocol (08-001607).

### Cell Lines, Antibodies and Reagents

Human pancreas ductal epithelial (HPDE) cells were obtained from M.S. Tsao (Ontario Cancer Institute, Ontario, Canada) and maintained as previously described [24]. All other cell lines were from the American Type Culture Collection (ATCC, Manassas, VA) and were maintained as suggested by the ATCC. The anti-p100/p52, anti-p50, anti-p65, anti-RelB, anti-cIAP1, anti-cIAP2, anti-TRAF3 (#4729) and anti-TRAF2 (#4724) antibodies for immunoblotting were from Cell Signaling Technology (Danvers, MA), anti-NIK antibody from Abcam (ab7204), anti-RelB (10x), anti-p52 (10x) and anti-pT559-NIK antibody from Santa Cruz (Santa Cruz, CA), anti-nucleolin, anti-HA, anti-FLAG (F7425), and anti-β-actin antibodies from Sigma-Aldrich (St Louis, MO), and anti-TRAF2 (IMG-5760) from Imgenex (San Diego, CA). Secondary HRP-linked anti-mouse or anti-rabbit antibodies were from Roche (Indianapolis, IN). MG-132 was from Millipore (Philadelphia, PA). 5-Fluorouracil (5-FU) was from Sigma and Gemcitabine from Lilly (Indianapolis, IN). Giemsa and Matrigel were from BD biosciences (Bedford, MA), agarose (Sea Plaque GTG) for soft agar assays from Lonza (Rockland, ME). Lipofectamine 2000 from Invitrogen (Carlsbad, CA) was used for transfection of Panc1, MiaPaca2 cells or HPDE cells. Cell lines stably expressing NIK or NIK-shRNA were generated by two week selection with Puromycin 400 ng/ml.

### Impedance-based Real-time Chemotaxis and Proliferation Assays

5,000 or 10,000 cells per well were seeded on E-Plates (proliferation assays) or Transwell CIM-plate 16 (motility assays) from Roche (Indianapolis, IN). For proliferation assays impedance for indicated times was continuously monitored in real-time using the xCELLigence RTCA DP instrument (Roche). For migration assays, after two hours of attachment, cell migration or invasion (coating of top well with 2 µg Matrigel) towards NIH-3T3 conditioned media was continuously monitored in real-time over indicated times using the xCELLigence RTCA DP instrument (Roche).

### MTT Assays

10,000 cells per well were seeded in 96 well plates and stimulated the next day as indicated. To measure cell viability, 10 µl of MTT in PBS (5 mg/ml) were added per well. Cells were incubated at 37°C for four hours and then 100 µl solubilization solution (10% SDS in 0.001 M HCl) was added over night. Plates were analyzed by reading at 600 nm using a SynergyHT plate reader (Bio Tek Instruments, Winooski, VT).

### 3D (3-dimensional) Cell Culture

Cell culture dishes were pre-coated with undiluted phenol red-free Matrigel (10 mg/ml). Then 10^4^ cells were suspended in a volume of 200 µl PBS and mixed with 100 µl of cold phenol red-free Matrigel (10 mg/ml) and added to each well of a 24 well plate. After the cell layer was set complete, culture media was added over the top. Media was changed every two days. Photos were taken after indicated days using a 10× magnification.

### Soft Agar Colony Formation Assays

The base agar (0.75% Sea Plaque GTG agarose in growth medium with FBS) was added into a 35 mm petridish (total volume: 2 ml). The agar was allowed to set for 30 minutes at 4°C and then incubated at 37°C in a cell culture incubator over night. For the top agar 1.5% Sea Plaque GTG agarose was melted and cooled to 37°C in a water bath. Cells were trypsinized and counted. For plating, 2× growth medium plus FBS were mixed with 1.5% agarose and 5,000 cells were added. Suspensions were gently mixed and plated on top of the base agar in a volume of 2 ml. Plates were incubated at 37°C in a cell culture incubator for 28 days. Colonies were fixed with methanol (15 minutes, room temperature) and stained with 2 ml Giemsa in PBS (1∶15) at RT for two hours. Cells were washed three times with PBS for 15 minutes and colonies were counted using ImagePro.

### DNA and Viral Constructs

The expression construct for FLAG-tagged NIK was generated using Myc-MAP3K14 from Origene Technologies (Rockville, MD) as a template and 5′-GCGGGATCCATGGCAGTGATGGGAATGGCCTGCCCA-3′ and 5′-GCGCTCGAGTTAAGCATAATCAGGAACATCATAAGGATAGGGCCTGTTCTCCAGCTGGCCATG-3′ as primers and by cloning into pcDNA4/TO (Invitrogen, Carlsbad, CA) via BamHI and XhoI. Mutagenesis was carried out using the QuikChange kit (Stratagene, La Jolla, CA). The T559D mutants were generated using above construct as a template and 5′-ATCCCTGGCGATGAGACC-3′ and 5′-GGTCTCATCGCCAGGGAT-3′ as primers. The lentiviral construct for expression of FLAG-tagged constitutively active NIK (NIK.T559D) was generated by cloning of human NIK.T559D into pLenti6.3/V5-TOPO vector according to the manufacturer’s instructions (Invitrogen). The lentiviral shRNA expression system to knock-down human NIK is commercially available from Sigma (SHDNA MISSION® shRNA Plasmid DNA; St. Louis, MO, USA). Sequences used were human MAP3K14 NM_003954.x-896 (labeled: NIK-shRNA1) and human MAP3K14 NM_003954.x-4315 (labeled: NIK-shRNA2). The ViraPower Lentiviral Expression System (Invitrogen) was used for an optimized mix of packaging plasmids to produce Lentivirus in 293FT cells. Adenovirus to express NF-κB2/p100 was purchased from Vector Biolabs (Philadelphia, PA). Adenovirus to express human TRAF2 (NM_021138) was purchased from Applied Biological Materials (Richmond, BC, Canada). The expression plasmid for HA-tagged ubiquitin was from E. Yeh (via Addgene; plasmid 18712; [25]), expression plasmids for FLAG- or GFP-tagged TRAF2 from H. Wajant (University Hospital Würzburg, Germany). The NF-κB-luciferase and renilla luciferase reporter constructs were described previously [26], [27].

### Immunoblotting, Immunoprecipitation and PAGE

Cells were washed twice with ice-cold PBS (140 mM NaCl, 2.7 mM KCl, 8 mM Na_2_HPO_4_, 1.5 mM KH_2_PO_4_, pH 7.2) and lysed with Triton buffer (50 mM Tris-HCl pH7.4, 1% Triton X-100, 150 mM NaCl, 5 mM EDTA pH 7.4) plus Protease Inhibitor Cocktail (PIC, Sigma-Aldrich, St. Louis, MO), or RIPA buffer (0.01 M NaHPO_4_ pH 7.2, 2 mM EDTA, 50 mM NaF, 150 mM NaCl, 0.1% SDS, 1% sodium deoxycholate, 1% Nonindet P-40) plus Protease Inhibitor Cocktail for total cell lysates. Lysates were vortexed and incubated on ice for 30 minutes. Following centrifugation (13,000 rpm, 15 minutes, 4°C) the supernatant was collected and subjected to SDS-PAGE (Western blotting) or proteins of interest were immunoprecipitated by one hour incubation with specific antibody (2 µg) followed by 30 minutes incubation with protein G-Sepharose (Amersham Biosciences). Immune-complexes were washed three times with TBS (50 mM Tris-HCl pH 7.4, 150 mM NaCl), resolved in 20 µl TBS and 2× Laemmli buffer and subjected to SDS-PAGE.

### Nuclear Extracts

Cells (10 cm dish, confluent) were rinsed twice with ice cold PBS. Cells were scratched in 1 ml lysis buffer (10 mM HEPES pH 7.9, 10 mM KCl, 0.1 mM EDTA, 0.1 mM EGTA, 1 mM DTT, 1 mM PMSF) and lysates incubated for 15 min on ice. 62.5 µl 10% NP-40 was added and samples were placed on a shaker (2 min at 4°C). Samples were spun down for 1 min at room temperature at 13,000 rpm and the supernatant collected (cytosolic fraction). The pellet was re-suspended in 100 µl high salt buffer (20 mM HEPES pH 7.9, 4 M NaCl, 1 mM EDTA, 1 mM EGTA, 1 mM DTT, 1 mM PMSF) followed by rough shaking for 20 min at 4°C, and centrifugation (15,000 rpm) for 5 min at 4°C. Supernatants were transferred to a new tube and protein concentration was determined.

### Electrophoretic Mobility Shift Assay (EMSA)

IRDye 700 NF-κB Consensus Oligonucleotide and Odyssey Infrared EMSA Kit were purchased from LI-COR Biosciences (Lincoln, NE). Unlabeled control oligonucleotides were purchased from Thermo Fisher Scientific (Rockford, IL). For shift assays, 20 µg of nuclear protein was incubated in 20 µl of a buffer containing 10 mM HEPES (pH 7.5), 50 mM KCl, 0.1 mM EDTA, 1 mM dithiotreitol, 0.1% NP-40 and 0.05 mg/ml poly(dI-dC) on ice. Unlabeled probe or 4 µg specific antibody (10×, Santa Cruz) was added for 1 hour on ice. Labeled probe was added 30 min before gel loading. Samples were resolved on a non-denaturing 5% polyacrylamide gel in 0.5× TBE. Imaging was performed on the Odyssey (LI-COR Biosciences) using the 700 nm channel.

### Reporter Gene Assays

Cells were transfected with NF-κB-luciferase reporter (NF-κB-luc, 3 µg), renilla luciferase reporter (0.1 µg), and the cDNA of interest (1 µg) per well of a 6 well plate. 24 hours after transfection, cells were washed twice with ice-cold PBS, scraped in 250 µl Passive Lysis Buffer (Promega) and centrifuged (13,000 rpm, 10 min, 4°C). Assays for luciferase activity were performed according to the Promega Luciferase assay protocol and measured using a Veritas luminometer (Symantec, Cupertino, CA). Luciferase activity of the NF-κB-reporter construct was normalized to renilla luciferase activity. Expression of proteins was controlled by immunoblot or RT-PCR analysis, as indicated.

### Quantitative Real Time PCR

The mRNA levels of NIK and TRAF2 were assessed using a 2 step quantitative reverse transcriptase-mediated real-time PCR (qPCR) method. Total RNA was isolated using the RNeasy kit (Qiagen, Frederick, MD) according to the manufacturer’s instructions and 500 ng total RNA from each cell line was converted to cDNA using the High Capacity cDNA Reverse Transcriptase kit (Applied Biosystems, Bedford, MA). Quantitative real-time PCR was performed with a 7900HT Fast real time thermocycler (Applied Biosystems) and the TaqMan Universal PCR Master Mix (Applied Biosystems) with 10 ng cDNA as template, appropriate probe/primer sets from Applied Biosystems (NIK: Hs00177695_m1; TRAF2: Hs00184192_m1; GAPDH: Hs99999905_m1) and the following thermocycler program: 95°C for 20 seconds; 40 cycles of 95°C for 1 second and 60°C for 20 second. Data were collected by a Prism 7900 sequence detector and analyzed with Sequence Detection System software (Applied Biosystems). Data were normalized to human GAPDH, and mRNA levels of NIK and TRAF2 in pancreatic cancer cell lines were calculated using the ΔΔ*C*_T_ method and plotted as relative fold to HPDE cells (normal control).

### Immunohistochemistry

Pancreatic Cancer TMAs (PA805a) were obtained from US Biomax and additional tissue tissue slides containing histologically-confirmed human pancreatic cancer and normal human pancreas tissue samples from the Mayo Clinic SPORE for Pancreatic Cancer Tissue Core Facility. Samples were deparaffinized (one hour at 60°C), dewaxed in xylene (five times for four minutes) and gradually rehydrated with ethanol (100%, 95%, 75%, twice with each concentration for three minutes). The rehydrated TMAs were rinsed in water and subjected to antigen retrieval in citrate buffer (pH 6.0) as described by the manufacturer (DAKO, Carpinteria, CA, USA). Slides were treated with 3% hydrogen peroxide (five minutes) to reduce endogenous peroxidase activity and washed with PBS containing 0.5% Tween 20. NIK, pT599-NIK and TRAF2 were detected using specific antibodies (anti-NIK at 1∶50, anti-pT599-NIK at 1∶50, anti-TRAF2 (Imgenex, IMG-5760) at 1∶2000) in PBS/Tween and visualized using the Envision Plus Dual Labeled Polymer Kit following the manufacturer’s instructions (DAKO). H&E staining was performed as previously described [26]. Images were captured using ImagePro software (Media Cybernetics, Bethesda, MD, USA).

### Statistical Analysis

Data are presented as mean ± SD. P values were acquired with the student’s *t*-test using Graph Pad software, and p<0.05 is considered statistically significant.

## Supporting Information

Figure S1 **Use of a second antibody directed against a different epitope to demonstrate that** **TRAF2 expression is downregulated in PDAC cell lines.** TRAF2 was immunoprecipitated and samples subjected to SDS-PAGE. Samples were transferred to nitrocellulose and analyzed by Western blot for expression of TRAF2 (anti-TRAF2, Imgenex, IMG-5760). Western blots for β-actin (anti-β-actin) served as control.
(PDF)

Figure S2 **Additional data to** **Figure 3B** **.** Nuclear extracts and cytosolic fractions of indicated cell lines (shown in Fig. 3B) were additionally analyzed by Western blotting for p65 and p50.
(PDF)

Figure S3 **Knockdown of NIK does not sensitize Panc1 cells to chemotherapeutics.** **A, B:** Panc1 cells stably-expressing control (scrambled) shRNA, NIK-shRNA1 or NIK-shRNA2 were seeded in 96 well plates. Cells were then treated with indicated doses of Gemcitabine (A) or 5-FU (B) for 48 hours. Living cells were determined with an MTT assay. All samples are normalized to untreated control cells (100% living cells). The error bar represents six experiments.
(PDF)

Figure S4 **Knockdown of NIK or expression of active NIK do not affect directed cell migration or invasion of PDAC cells.** **A–D:** Panc1 (A, B) or MiaPaca2 cells (C, D) stably expressing control (scrambled) shRNA, NIK-shRNA1 or NIK-shRNA2 (A, C), or lentivirally infected with control virus or NIK.T559D mutant (B, D) were seeded in Transwell CIM-plate 16 plates. After attachment, cell migration towards NIH-3T3 conditioned media was continuously monitored in real-time over indicated times using a xCELLigence RTCA DP instrument. Error bars (gray) represent three experiments.
(PDF)
