# Supplementary material for: Downregulation of TRAF2 Mediates NIK-Induced Pancreatic Cancer Cell Proliferation and Tumorigenicity
Source: PLoS One. 2013 Jan 3;8(1):e53676. doi: 10.1371/journal.pone.0053676 (PMC3536768; doi:10.1371/journal.pone.0053676)
Supplement: Figure S1 — Use of a second antibody directed against a different epitope to demonstrate that TRAF2 expression is downregulated in PDAC cell lines. TRAF2 was immunoprecipitated and samples subjected to SDS-PAGE. Samples were transferred to nitrocellulose and analyzed by Western blot for expression of TRAF2 (anti-TRAF2, Imgenex, IMG-5760). Western blots for β-actin (anti-β-actin) served as control. (PDF) [file pone.0053676.s001.pdf]

Supplemental Figure S1

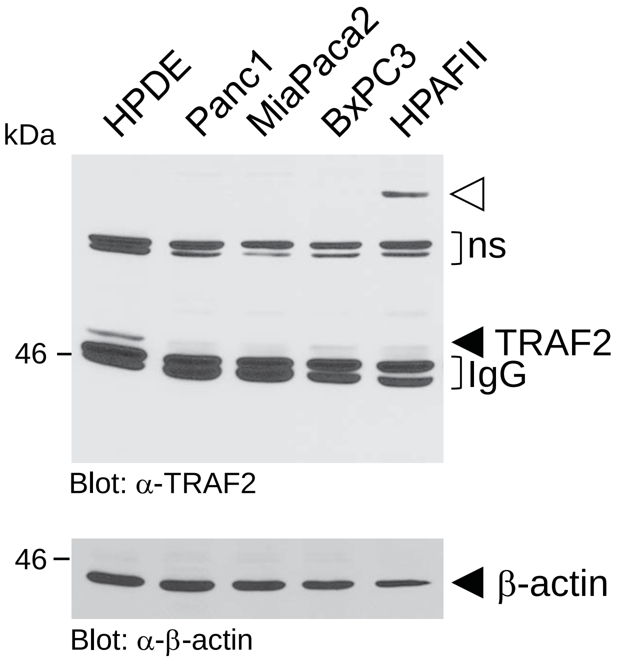

Supplemental Fig. S1: **Use of a second antibody directed against a different epitope to demonstrate that TRAF2 expression is downregulated in PDAC cell lines.** TRAF2 was immunoprecipitated and samples subjected to SDS-PAGE. Samples were transferred to nitrocellulose and analyzed by Western blot for expression of TRAF2 (anti-TRAF2, Imgenex, IMG-5760). Western blots for β-actin (anti-β-actin) served as control.
