# Supplementary material for: Downregulation of TRAF2 Mediates NIK-Induced Pancreatic Cancer Cell Proliferation and Tumorigenicity
Source: PLoS One. 2013 Jan 3;8(1):e53676. doi: 10.1371/journal.pone.0053676 (PMC3536768; doi:10.1371/journal.pone.0053676)
Supplement: Figure S2 — Additional data to Figure 3B . Nuclear extracts and cytosolic fractions of indicated cell lines (shown in Fig. 3B) were additionally analyzed by Western blotting for p65 and p50. (PDF) [file pone.0053676.s002.pdf]

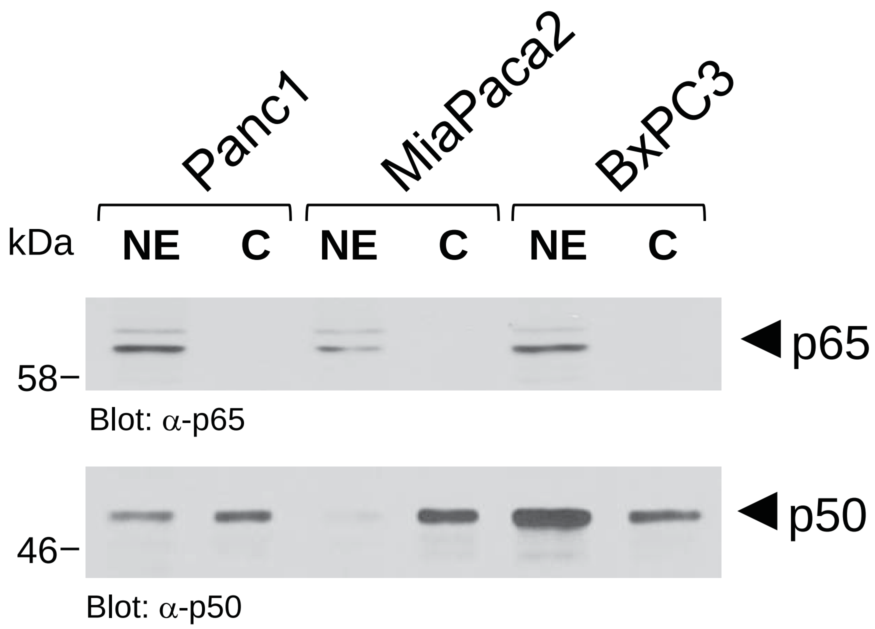

Supplemental Fig. S2: **Additional data to Figure 3B.** Nuclear extracts and cytosolic fractions of indicated cell lines (shown in Fig. 3B) were additionally analyzed by Western blotting for p65 and p50.
