# Supplementary material for: Downregulation of TRAF2 Mediates NIK-Induced Pancreatic Cancer Cell Proliferation and Tumorigenicity
Source: PLoS One. 2013 Jan 3;8(1):e53676. doi: 10.1371/journal.pone.0053676 (PMC3536768; doi:10.1371/journal.pone.0053676)
Supplement: Figure S3 — Knockdown of NIK does not sensitize Panc1 cells to chemotherapeutics. A, B: Panc1 cells stably-expressing control (scrambled) shRNA, NIK-shRNA1 or NIK-shRNA2 were seeded in 96 well plates. Cells were then treated with indicated doses of Gemcitabine (A) or 5-FU (B) for 48 hours. Living cells were determined with an MTT assay. All samples are normalized to untreated control cells (100% living cells). The error bar represents six experiments. (PDF) [file pone.0053676.s003.pdf]

Supplemental Figure S3

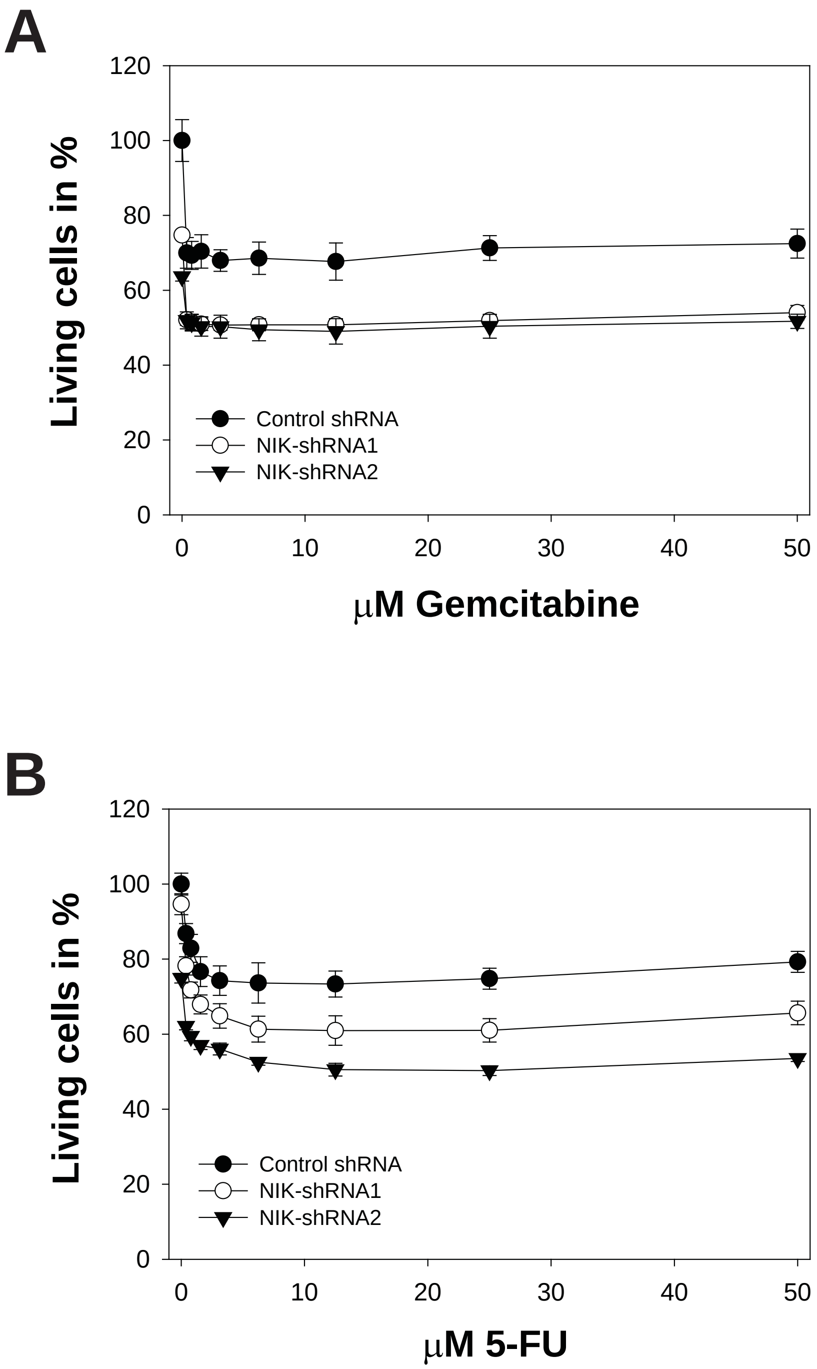

Supplemental Fig. S3: **Knockdown of NIK does not sensitize Panc-1 cells to chemotherapeutics. A, B:** Panc1 cells stably-expressing control (scrambled) shRNA, NIK-shRNA1 or NIK-shRNA2 were seeded in 96 well plates. Cells were then treated with indicated doses of Gemcitabine (A) or 5-FU (B) for 48 hours. Living cells were determined with an MTT assay. All samples are normalized to untreated control cells (100% living cells). The error bar represents six experiments.
