# Supplementary material for: Downregulation of TRAF2 Mediates NIK-Induced Pancreatic Cancer Cell Proliferation and Tumorigenicity
Source: PLoS One. 2013 Jan 3;8(1):e53676. doi: 10.1371/journal.pone.0053676 (PMC3536768; doi:10.1371/journal.pone.0053676)
Supplement: Figure S4 — Knockdown of NIK or expression of active NIK do not affect directed cell migration or invasion of PDAC cells. A–D: Panc1 (A, B) or MiaPaca2 cells (C, D) stably expressing control (scrambled) shRNA, NIK-shRNA1 or NIK-shRNA2 (A, C), or lentivirally infected with control virus or NIK.T559D mutant (B, D) were seeded in Transwell CIM-plate 16 plates. After attachment, cell migration towards NIH-3T3 conditioned media was continuously monitored in real-time over indicated times using a xCELLigence RTCA DP instrument. Error bars (gray) represent three experiments. (PDF) [file pone.0053676.s004.pdf]

Supplemental Figure S4

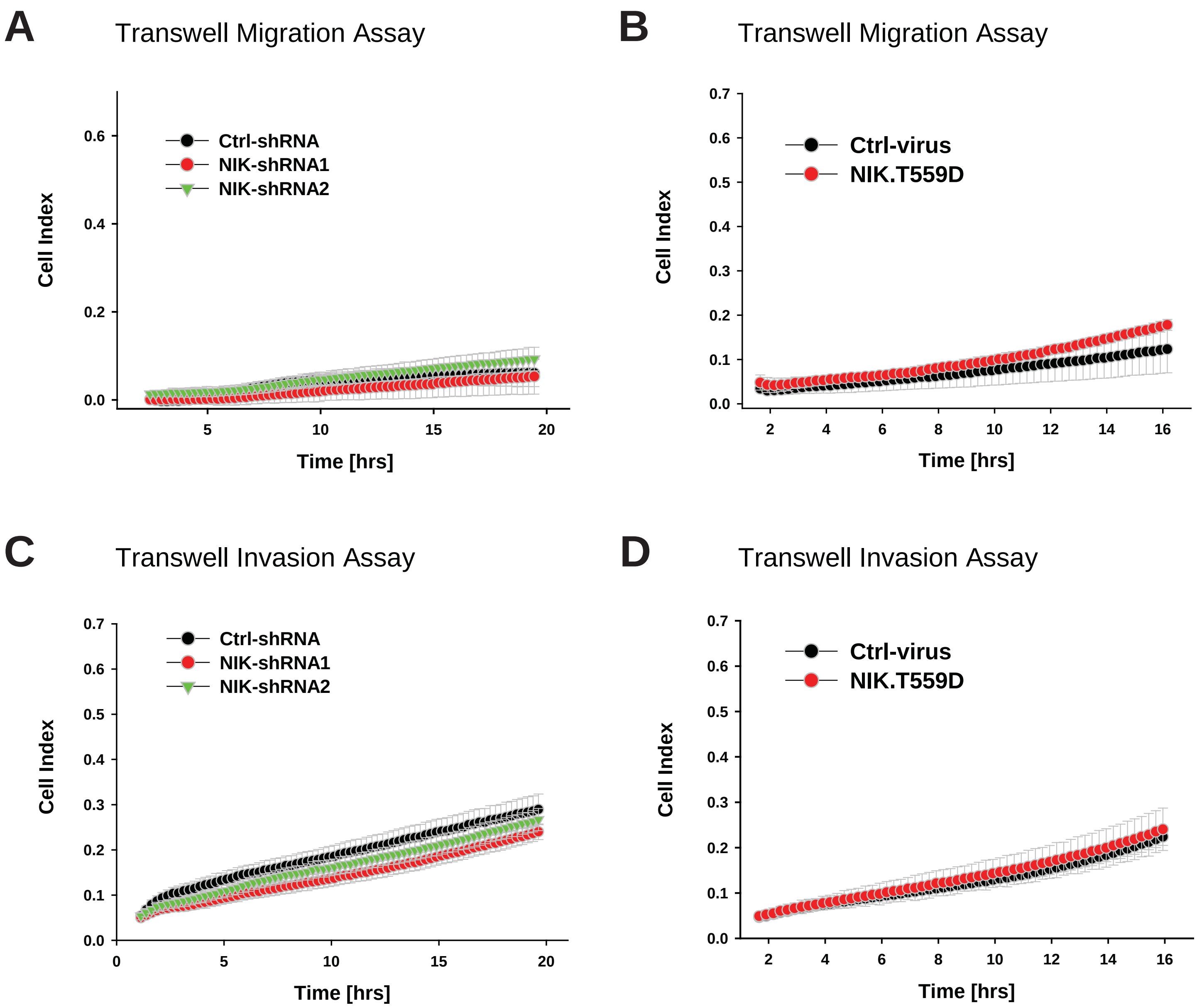

Supplemental Fig. S4: **Knockdown of NIK or expression of active NIK do not affect directed cell migration or invasion of PDAC cells. A-D:** Panc1 (A, B) or MiaPaca2 cells (C, D) stably-expressing control (scrambled) shRNA, NIK-shRNA1 or NIK-shRNA2 (A, C), or lentivirally-infected with control virus or NIK.T559D mutant (B, D) were seeded in Transwell CIM-plate 16 plates. After attachment, cell migration towards NIH-3T3 conditioned media was continuously monitored in real-time over indicated times using a xCELLigence RTCA DP instrument. Error bars (grey) represent three experiments.
